# Correction: Prevalence of mental health problems among stranded international students during the COVID-19 pandemic

**DOI:** 10.1186/s40359-022-00939-w

**Published:** 2022-10-21

**Authors:** Shandana Iftikhar, Garon Perceval, Yining Fu, Chuan Zhou, Yongguo Cao

**Affiliations:** 1 School of Education, Soochow University, Room No. 5627, Building 1005, Dushu lake campus, Suzhou, China; 2 UQ Centre for Clinical Research, The University of Queensland, St Lucia, Australia

## BMC psychology (2022) 10:211

10.1186/s40359-022-00917-2.

Following publication of the original article [1], it came to the journal’s attention that references 7, 8, 11, and 39 had been erroneously replaced by the following text: “Elsevier has created a COVID-19 resource centre with free information in English and Mandarin on the novel coronavirus COVID-19. The COVID-19 resource centre is hosted on Elsevier Connect, the company’s public news and information”. The references have since been corrected in the published article.

